# Physician Trainees' Perception of Cannabidiol Use in Medicine: A Survey Study

**DOI:** 10.7759/cureus.47228

**Published:** 2023-10-17

**Authors:** Alexandra Boyd, Ajith Malige, Orr Limpisvasti

**Affiliations:** 1 Orthopaedic Surgery, Cedars-Sinai Kerlan-Jobe Institute, Los Angeles, USA

**Keywords:** cannabidiol, chronic pain, stigma in health care, acute pain management, medical student training, orthopaedic surgery, cannabidiol (cbd)

## Abstract

Purpose: Given the ongoing national opiate crisis, physicians have been challenged with mitigating the risk of opiate dependence in their patients. With current physician efforts to mitigate the risks of treating pain with opioid prescriptions, this study evaluates medical students' and residents' understanding and perceptions regarding cannabidiol (CBD) in current medical care and their future medical practice.

Methods: Orthopedic residents from all American programs and medical students from 50 medical schools, regardless of training year or future specialty plans, were eligible to participate in this survey-based study administered from December 2022 to March 2023. The surveys ask questions about demographic information, what education they receive on CBD utilization in medicine, thoughts on CBD effectiveness in pain control, and future plans on utilizing CBD.

Results: A total of 55 residents (1.4%) and 53 medical students (5.1%) responded. Trainees in CBD-legal states were more likely to work with physicians who use CBD in their practice. Most trainees, regardless of location, believe CBD use has a stigma attached to it. Many responders were concerned about the role of CBD in pain control. Finally, most trainees believed that CBD is easy to access if desired and is affordable to purchase.

Conclusion: The trajectory of CBD use in the United States indicates that the therapeutic benefits of CBD will be targeted, and future physicians are not always provided adequate educational opportunities to learn about its potential medical uses. Continued training as well as interactions with patients may help decrease the stigma surrounding medical CBD use and help solidify its therapeutic use in pain control.

## Introduction

Given the ongoing national opiate crisis, physicians have been challenged with mitigating the risk of opiate dependence in their patients. Multimodal analgesia has thus become the standard of pain management. Unfortunately, these non-opioid alternatives are not always successful in adequately controlling patient pain. Because of this, patients are trying alternative medications, such as cannabidiol (CBD)-containing products, in hopes of managing both acute and chronic pain. Marijuana consists of tetrahydrocannabinol (THC) and CBD. THC produces psychotropic effects through dopamine excitation of cannabinoid receptors in the central and peripheral nervous systems. CBD, in contrast, can potentially provide analgesia without the psychoactive effects due to its effect on opioid receptors [1,2].

Recent studies have shown that CBD has been a successful adjunct for patients in need of pain management in both operative and non-operative treatment. Multiple studies highlight the benefits of CBD use in treating postoperative and arthritic pain, noting improved physical function, improved sleep quality and quality of life, and reduced NSAID, acetaminophen, and opioid use [3,4]. However, some studies have found that the use of CBD did not reduce pain or opioid consumption in arthroplasty patients [5,6]. In addition, CBD products, including Epidiolex®, have been approved by the Food and Drug Administration (FDA) as safe and effective treatments for multiple medical pathologies. However, CBD use in medicine remains controversial due to a paucity of reported data regarding postoperative complications (despite most reports documenting a low overall complication rate), variability in the content of CBD products, potential interactions with other treatment modalities, and a higher risk of opioid misuse [7-9]. While different forms of CBD medications (oral, oils, drops, tinctures, etc.) exist, the relative effectiveness of one form over the other has not been well-researched. Instead, the most effective modes are based on patient preference and ease of use rather than patient pathology.

Currently, there remains a stigma about CBD use in the general population and even more concern about its inclusion in medical care. Despite the use and sales of CBD products in multiple states, studies have yet to definitely elucidate physicians' and future physicians' sentiments regarding the addition of CBD use in pain control regimens. Chin et al. surveyed the members of the Orthopaedic Trauma Association and found that 88% of trauma surgeons did not believe they were knowledgeable in the mechanism of CBD, but 73% believed CBD has a role to play in the treatment of postoperative pain [10]. The same type of study, however, has not been completed among physician trainees, the next generation of healthcare providers who are at the forefront of patient care and a fundamental resource of information for patients during training. With current physician efforts to mitigate the risks of treating pain with opioid prescriptions, we set out to evaluate medical students' and residents' understanding and perceptions regarding CBD in current medical care and their future medical practice. The receptiveness to CBD in future medical professionals may predict increased inclusion of CBD in medical education and training, research, and clinical use.

## Materials and methods

Approval for this cross-sectional survey study was obtained from the Cedars-Sinai Institutional Review Board (STUDY00002418). From December 2022 to March 2023, all orthopedic residencies in the country (3,828 total residents) and 109 medical schools (1,042 total medical students), with at least one from every state, were asked to participate. Each program was contacted by email three times to distribute surveys to their trainees. The first email was sent in December, with two reminder emails sent six weeks apart. All orthopedic residents and medical students, regardless of year of training or future specialty plans, were eligible to participate.

The survey aimed to identify physician trainees' understanding, previous training, and perceptions of the use of CBD in pain control regimens [11]. While there are no similar studies surveying trainees, our survey was constructed based on previous CBD survey studies [12-14]. The 18-question anonymous survey was administered through RedCap (Vanderbilt University, Nashville, TN) [15,16]. Both the resident (Appendix 1) and medical student surveys (Appendix 2) ask questions about basic demographic information, including whether the responder is training in a state where CBD use is legal or not, what education they receive on CBD utilization in medicine, thoughts on the effectiveness of CBD in pain control, and whether they plan on utilizing CBD in their future practice.

Data were recorded in RedCap. Survey answers were analyzed utilizing descriptive statistics and statistical tests as appropriate (chi-square or Fisher’s exact tests as appropriate) to determine differences in responses between CBD legal and non-legal states (IBM SPSS Statistics for Windows, version 23, IBM Corp., Armonk, NY). For all analyses, statistical significance was set at p < 0.05.

## Results

Resident survey

In total, 55 residents (1.4%) responded to the survey. Fifteen (27.3%) are postgraduate year (PGY) 1 trainees, six (10.9%) are PGY-2, eight (14.5%) are PGY-3, 10 (18.2%) are PGY-4, and 11 (20.0%) are PGY-5 trainees. Most responders are training at academic hospitals (n = 50, 90.9%). Eleven participants (20.0%) plan on entering a sports medicine fellowship after residency, and an additional 11 (20.0%) plan on entering a joint fellowship. Additionally, most trainees reside in a state where CBD use is legal (n = 31, 56.4%) (Table 1).

**Table 1:** Demographic information of orthopedic surgery resident responders. Data have been represented as mean ± SD and N (%). PGY = postgraduate year; CBD = cannabidiol; F&A = foot and ankle; S&E = shoulder and elbow; Other = multiple choices or unsure; CBD use legal = responder is from a state where CBD use is legal.

		Residency year					Total
		PGY-1	PGY-2	PGY-3	PGY-4	PGY-5	
Average age (years)		27.9 ± 1.2	30.1 ± 4.2	31.6 ± 2.1	30.4 ± 1.4	32.2 ± 1.7	30.1 ± 2.6
Training center	Academic	15 (27.3%)	6 (10.9%)	8 (14.5%)	10 (18.2%)	11 (20.0%)	50 (90.9%)
	Community	2 (3.6%)	2 (3.6%)	0 (0.0%)	1 (1.8%)	0 (0.0%)	5 (9.1%)
Future fellowship plan	Sports	3 (5.5%)	2 (3.6%)	1 (1.8%)	1 (1.8%)	4 (7.3%)	11 (20.0%)
	Joints	1 (1.8%)	2 (3.6%)	2 (3.6%)	4 (7.3%)	2 (3.6%)	11 (20.0%)
	Trauma	2 (3.6%)	0 (0.0%)	1 (1.8%)	2 (3.6%)	0 (0.0%)	5 (9.1%)
	Hand	3 (5.5%)	0 (0.0%)	2 (3.6%)	1 (1.8%)	2 (3.6%)	8 (14.5%)
	F&A	0 (0.0%)	0 (0.0%)	0 (0.0%)	1 (1.8%)	0 (0.0%)	1 (1.8%)
	S&E	0 (0.0%)	0 (0.0%)	0 (0.0%)	1 (1.8%)	1 (1.8%)	2 (3.6%)
	Spine	1 (1.8%)	3 (5.5%)	1 (1.8%)	1 (1.8%)	2 (3.6%)	8 (14.5%)
	Other	7 (12.7%)	1 (1.8%)	1 (1.8%)	0 (0.0%)	0 (0.0%)	9 (16.4%)
CBD use legal	Yes	11 (20.0%)	4 (7.3%)	3 (5.5%)	7 (12.7%)	6 (10.9%)	31 (56.4%)
	No	6 (10.9%)	4 (7.3%)	5 (9.1%)	4 (7.3%)	5 (9.1%)	24 (43.6%)
Total		17 (30.9%)	8 (14.5%)	8 (14.5%)	11 (20.0%)	11 (20.0%)	55 (100%)

**Figure 1:**
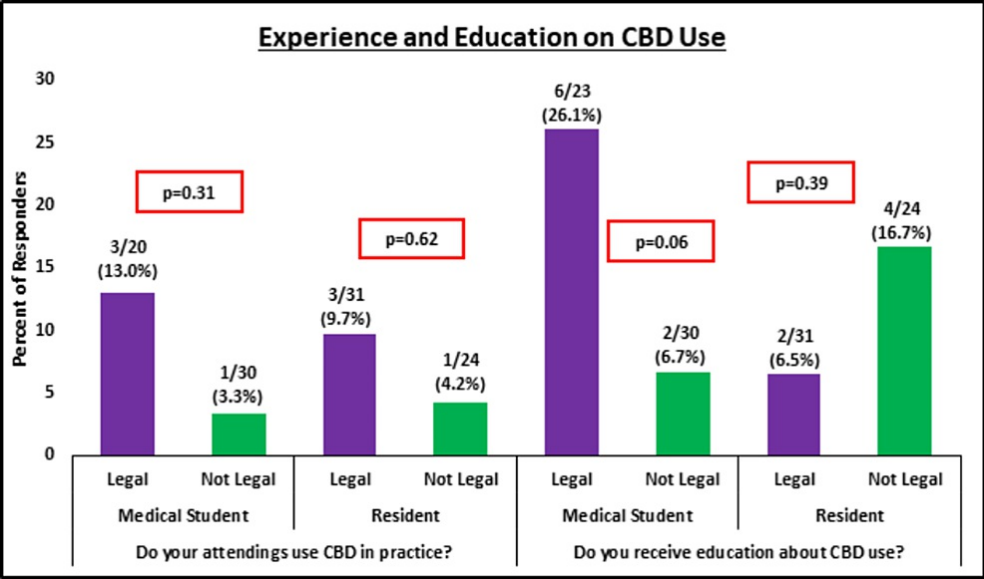
Experience and education on CBD use. CBD = cannabidiol; Legal = responders are from states where CBD use is legal; Not legal = responders are from states where CBD use is not legal.

**Figure 2:**
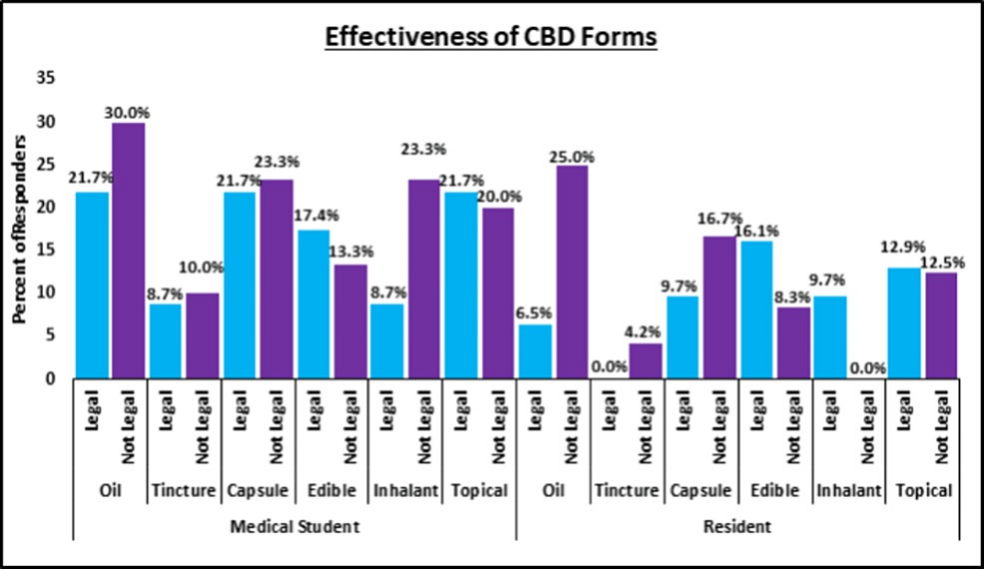
Effectiveness of CBD forms. CBD = cannabidiol; Legal = responders are from states where CBD use is legal; Not legal = responders are from states where CBD use is not legal.

Residents training in states where CBD use is legal were more likely to work with physicians who use CBD in their practice, even though the difference between cohorts was not significant (p = 0.62). Interestingly, a higher proportion of residents in CBD non-legal states noted receiving education about CBD use, even though this difference between cohorts was not significant either (p = 0.39) (Figure 1). A high percentage of residents in CBD-legal (n = 19, 61.2%) and non-legal (n = 18, 75.0%) states responded that they believed the use of CBD has a stigma attached to it. Higher proportions of residents from CBD-legal states thought that edible, inhalant, and topical forms for CBD were effective, while higher proportions from CBD non-legal states thought that oils, tinctures, and capsules were effective (Figure 2).

Regardless of location, less than 10% of residents were concerned about the effect CBD has on bone and wound healing. While a higher proportion of residents from CBD-legal states were concerned about the true effect CBD would have in helping to control postoperative pain, this difference between cohorts was not significant (5/31 (16.1%) vs. 1/24 (4.2%), p = 0.21) (Table 2). Finally, residents in CBD-legal states were just as likely as those from non-legal states to think that CBD is easy to access (24/31 (77.4%) vs. 16/24 (75.0%), p = 0.83) and it is affordable to purchase if desired (20/31 (64.5%) vs. 15/24 (62.5%), p = 0.88).

**Table 2:** Demographic information of medical student responders. Data have been represented as mean ± SD and N (%). CBD = cannabidiol; Primary care = family medicine, internal medicine, and pediatrics; Surgery = general surgery, obstetrics and gynecology, orthopedic surgery, otolaryngology, neurosurgery, and plastic surgery; EM = emergency medicine; CBD use legal = responder is from a state where CBD use is legal.

		Medical school year			Total
		MS1	MS2	MS3/MS4	
Average age (years)		24.2 ± 2.3	25.0 ± 1.5	27.7 ± 3.2	24.9 ± 2.8
Training center	Academic	35 (66.0%)	5 (9.4%)	10 (19.9%)	50 (94.3%)
	Community	2 (3.8%)	1 (1.9%)	0 (0.0%)	3 (5.7%)
Future residency plans	Anesthesiology	1 (1.9%)	0 (0.0%)	1 (1.9%)	2 (3.8%)
	Dermatology	1 (1.9%)	0 (0.0%)	0 (0.0%)	1 (1.9%)
	EM	2 (3.8%)	0 (0.0%)	0 (0.0%)	2 (3.8%)
	Primary care	11 (20.8%)	5 (9.4%)	2 (3.8%)	18 (34.0%)
	Surgery	16 (30.2%)	1 (1.9%)	2 (3.8%)	24 (45.3%)
	Psychiatry	3 (5.7%)	0 (0.0%)	0 (0.0%)	3 (5.7%)
	Radiology	1 (1.9%)	0 (0.0%)	0 (0.0%)	1 (1.9%)
	Transition year	2 (3.8%)	0 (0.0%)	0 (0.0%)	2 (3.8%)
CBD use legal	Yes	9 (17.0%)	5 (9.4%)	9 (17.0%)	23 (43.4%)
	No	28 (52.8%)	1 (1.9%)	1 (1.9%)	30 (56.6%)
Total		37 (69.8%)	6 (11.3%)	10 (18.9%)	53 (100%)

Medical student survey

In total, 53 medical students (5.1%) responded to the survey. Thirty-seven students (69.8%) were first-year medical students, six (11.3%) were second-year students, and 10 (18.9%) were third-year and fourth-year students. Most responders were studying at an academic institution (n = 50, 94.3%). Eighteen students (34.0%) were planning on entering primary care specialty residencies (three family medicine, 11 internal medicine, and four pediatrics), while 24 (45.3%) were planning on entering surgical specialties (five general surgery, two neurosurgery, three obstetrics and gynecology, six orthopedic surgery, four otolaryngology, and four plastic surgery). In addition, most responders (n = 30, 56.6%) were from states where CBD use is not legal (Table 2).

Medical students training in states where CBD use is legal were understandably more likely to work with physicians who use CBD in their practice, even though the difference between cohorts was not significant (p = 0.31). While a higher proportion of CBD legal-state medical students receive education on CBD use, this difference was trending toward significance (26.1% vs. 6.7%, p = 0.06) (Figure 1). A higher percentage of students in CBD-legal (n = 18, 78.2%) and non-legal (n = 25, 83.3%) states responded that they believe CBD use does have a stigma attached to it. Higher proportions of medical students from CBD-legal states thought edibles and topical CBD forms were effective while higher proportions from CBD non-legal states thought oil, tincture, capsule, and inhalant CBD forms were effective (Figure 2).

Regardless of the location of training, very few students thought that CBD would affect bone healing and wound healing postoperatively, while over a quarter were concerned about the true effect CBD would have on pain control (Table 3). Finally, medical students in CBD-legal states were more likely than those from non-legal states to think that CBD is easy to access (16/23 (69.6%) vs. 12/30 (40.0%), p = 0.03) and just as likely to think it is affordable to purchase if desired (13/23 (56.5%) vs. 13/30 (43.3%), p = 0.34).

**Table 3:** Medical students' and residents' perceptions of the effect of cannabidiol on surgical outcomes. Data have been represented as N (%). P-values are significant at p < 0.05. Also, include the value at which the p-value is considered significant (p < 0.05, p < 0.001).

Affected surgical outcome	Medical students			Residents		
	Legal	Not legal	p-value	Legal	Not legal	p-value
Bone/wound healing	1 (4.3%)	2 (6.7%)	1.00	3 (9.7%)	2 (8.3%)	1.00
Pain control	8 (34.8%)	8 (26.7%)	0.76	5 (16.1%)	1 (4.2%)	0.21
Total	23	30		31	24	

## Discussion

As the public gravitates toward holistic ways of managing their health, the attribution of CBD as a natural product and its increased availability has bolstered its popularity and rendered CBD as a generally recognized supplement. With the progression of the opioid epidemic, prescriber vigilance for opioid misuse and search for alternative solutions for effective pain control is of increasing priority. It is the physician’s responsibility to keep pace with the changing landscape of pain management using CBD and to ensure future healthcare providers are educated and prepared for the discussion of CBD use in medicine.

As of 2023, all 50 states have laws detailing CBD legality, either demarcating it as fully legal or only conditionally legal for specific medical conditions [17]. Resident respondents to our survey mainly resided in CBD-legal states (56.4%), while most medical student participants (56.6%) did not. Trainees in CBD-legal states had an expectedly higher rate of gaining experience with physicians who utilize CBD in their practice. In addition, more medical students in CBD-legal states reported receiving education about CBD use. Unexpectedly, more residents in CBD non-legal states reported receiving education about CBD in their training, although not significant. This could be a product of the small sample size and the fact that trainees sometimes move to different states throughout their medical education. Previous studies highlight a general lack of training for future physicians regarding CBD discussion with patients [18], with physicians across the country expressing concern for the lack of CBD education for trainees [19,20].

Surveyed medical students and residents, regardless of CBD legality in their state, revealed concern for the stigma associated with CBD and its efficacy in pain control. Marijuana is federally classified as a Schedule I controlled substance by the Drug Enforcement Administration, with “no currently accepted medical use and a high potential for abuse” [21], but its state-level regulation is heterogeneous. However, medical marijuana and CBD should be distinguished as two separate entities. Labeling CBD as medical marijuana, an illegal drug for recreational purposes, makes it difficult for patients who may benefit from medicinal CBD to discuss it with their physicians in fear of its criminalization and being categorized as a “drug user” [22].

Even though the number of studies evaluating CBD safety and efficacy has increased recently, an overall lack of data to support CBD use in the treatment of pain leads physicians to be cautious when discussing with patients [23]. Other therapeutic benefits in the treatment of anxiety, depression, epilepsy, insomnia, and muscle disorders have been associated with CBD use as well. In addition, association of CBD with transaminase elevations, sedation, infection, anemia, and inhibition of several cytochromes P450, along with potential for drug-drug interactions or drug adverse events, reinforces the need for further research and understanding of CBD [14]. The need to obtain FDA approval for CBD-based medications before running clinical trials on their effectiveness presents a challenging and long road for any future studies.

Residents and medical students, regardless of their state’s CBD legality, considered edible and topical forms of CBD to be most effective in pain management. Topical CBD has been used safely to decrease pain and subjective time to wound healing time in two case series in humans [24,25], with one clinical study showing improvement in both wound appearance and symptom relief [26]. However, there is a dearth of clinical evidence for the efficacy and safety of topical CBD, even though its negligible absorption and evasion of first-pass metabolism and gastrointestinal administration should cause less concern for systemic side effects [27]. Inhalants were identified as most effective only by CBD-legal state residents and CBD-non-legal state medical students, which aligns with the fact that inhaled CBD avoids metabolism by the liver and has a quicker onset of action. Additionally, trainee responders shared little concern over CBD’s effect on wound healing and bone formation, a perception backed by previous studies that link the use of CBD to enhanced bone formation in fracture healing and critical-sized bone defects in a rat model [28].

The vast majority of residents and medical students, regardless of CBD legality in their state, believed that CBD was both affordable and accessible for patients. However, the cost of CBD is US$3.68-14.71 per 100 mg [29]. With extended use of CBD for pain management, cost has the potential to play a major role in patient willingness to use CBD instead of another, insurance-covered option, like opioids. In addition, although trainees believed CBD was accessible, the FDA has issued warnings for illegal selling of unapproved over-the-counter CBD products for pain relief [30]. There is only one FDA-approved prescription containing CBD. Physician awareness of cost and non-FDA-approved CBD accessibility can influence recommendations made to their patients and the willingness of patients to consider these options.

Limitations of this study are those inherent to a survey study. Many studies, including our current study, that analyze perceptions surrounding medicinal CBD use in pain management have sample sizes that are too small to reach significance. This lack of ample data increases non-responder bias and fails to provide sufficient evidence that supports larger clinical trials. The accuracy of responder answers, especially whether responders are from CBD legal or non-legal states, cannot be validated either. Surveys were also sent to respective program coordinators, with no way of knowing which trainees received the survey to complete, possibly affecting response rates. Finally, while trainees from every state were asked to participate, responders were probably not evenly distributed across the country. A larger study capturing trainees from all environments can help strengthen future studies and decrease responder bias.

## Conclusions

The trajectory of CBD use in the United States indicates that the therapeutic benefits of CBD will be targeted, and future physicians are not always provided adequate educational opportunities to learn about its potential medical uses. As CBD use continues to become more prevalent in our society, it is up to healthcare professionals to identify any potential roles of CBD in health care as well as clarify its role in each patient population. Continued training as well as interactions with patients may help decrease the stigma surrounding medical CBD use and help solidify its therapeutic use in pain control.

## Figures and Tables

**Figure 3:**
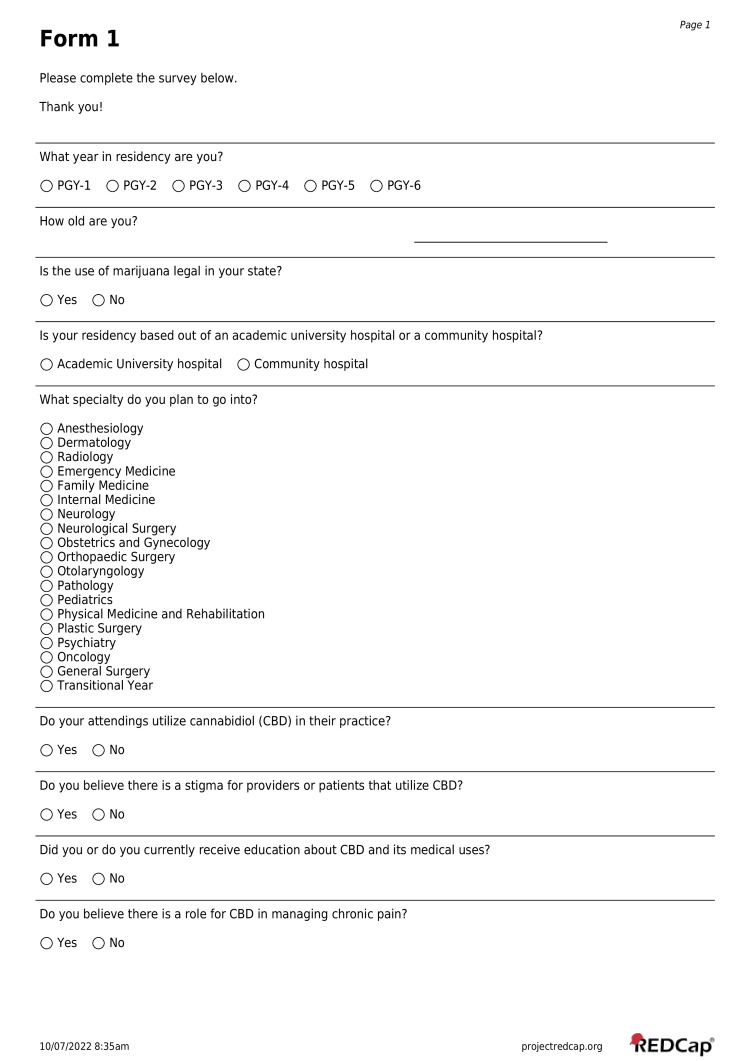
Orthopedic resident survey

**Figure 4:**
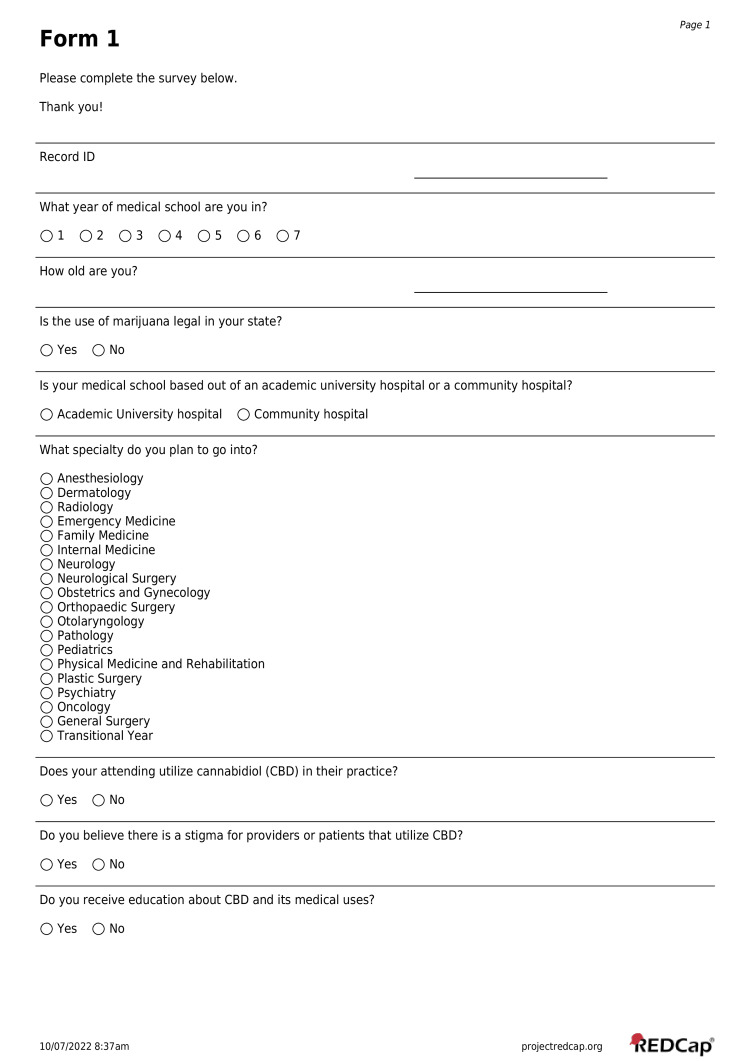
Medical student survey

## Appendices

Appendix 1

Appendix 2
